# A CXCR4/PD-L1 Bispecific Nanobody Engineered for Tumor Microenvironment Retention Mediates Sustained Synergy with Chemotherapy via Remodeling Immunity in TNBC

**DOI:** 10.3390/ph19081288

**Published:** 2026-08-14

**Authors:** Shuyi Xu, Hai Hu, Yifan Li, Jiawei Zhang, Lei Wang, Pameila Paerhati, Wenxin Bao, Yanlin Bian, Jianwei Zhu, Mingyuan Wu

**Affiliations:** 1 Engineering Research Center of Cell & Therapeutic Antibody, Ministry of Education, School of Pharmacy, Shanghai Jiao Tong University, Shanghai 200240, China; shuyixu@sjtu.edu.cn (S.X.);; 2 BioKu Biopharmaceuticals Co., Ltd., Shanghai 200240, China

**Keywords:** TNBC, bispecific nanobody, paclitaxel, immune microenvironment

## Abstract

**Background**: The efficacy of chemotherapy in triple-negative breast cancer (TNBC) is limited by intrinsic resistance and the tumor microenvironment (TME). Accumulating evidence reveals a mechanistic connection between programmed death-ligand 1 (PD-L1) and c-x-c motif chemokine receptor 4 (CXCR4), which dominate stroma barriers, immune escape, and cancer metastasis. Earlier studies have shown that dual suppression of c-x-c motif ligand 12 (CXCL12)/CXCR4 and programmed cell death-1 (PD-1)/PD-L1 pathways regulates extracellular matrix (ECM) deposition, activation of cancer-associated fibroblasts (CAFs), and epithelial–mesenchymal transition (EMT) of pancreatic cancer cells. **Methods**: We combined BsNb PX4, a bispecific nanobody targeting PD-L1 and CXCR4, with paclitaxel or gemcitabine in multiple tumor cell lines and human peripheral blood mononuclear cell (hPBMC)-reconstituted xenograft mouse models. Antitumor activity was assessed by CCK-8, flow cytometry, and ELISA, and immune cell infiltration and TME remodeling were examined by immunofluorescence, immunohistochemistry, cytokine assays, and RNA-seq. **Results**: In MDA-MB-231 cells, BsNb PX4 synergistically enhanced paclitaxel-induced growth inhibition and apoptosis via G_2_/M cycle arrest. This combinatorial strategy profoundly remodeled tumor immunity by expanding CD8^+^ T cells and depleting Foxp3^+^ CD4^+^ regulatory T cells (Tregs), while concurrently restoring T-cell cytotoxicity and skewing the cytokine balance toward an antitumor state, with elevated IFN-γ and reduced TGF-β1. Notably, compared with paclitaxel monotherapy, the combination significantly elevated intratumoral CD8^+^ T-cell infiltration, decreased Treg abundance, and exerted robust inhibitory effects on tumor growth and metastasis in humanized TNBC xenografts. **Conclusions**: These findings reveal that dual blockade of PD-L1 and CXCR4 acts synergistically with chemotherapy by triggering tumor cell apoptotic effects and reversing the immunosuppressive microenvironment, thereby emerging as a promising therapeutic strategy for TNBC.

## 1. Introduction

Triple-negative breast cancer, defined by the absence of estrogen receptor (ER), progesterone receptor (PR), and human epidermal growth factor receptor 2 (HER2), remains one of the most aggressive and therapeutically challenging breast cancer subtypes [1]. Despite accounting for only 15–20% of all breast cancers [2], TNBC contributes an extremely high mortality rate due to intrinsic therapeutic resistance and strong metastatic capacity [3]. Anthracycline–taxane regimens remain the cornerstone of TNBC management [4], while platinum-based agents enhance the pathological complete response (pCR) to neoadjuvant chemotherapy, particularly in *BRCA* gene-mutated patients [5]. In addition, gemcitabine constitutes a mainstay of metastatic TNBC therapy, routinely synergizing with platinum [6]. Nevertheless, only about 30% of TNBC patients exhibit initial chemosensitivity, with most developing acquired resistance, leading to more than 50% of patients relapsing within the first 3–5 years [7]. Drug resistance arises from both intrinsic survival signals within tumor cells and extrinsic protection provided by the immunosuppressive microenvironment [8,9].

Recent evidence has highlighted the pivotal role of the tumor microenvironment in chemoresistance. The TME of TNBC exhibits high heterogeneity, characterized by immune-suppressive cell infiltration such as Tregs and CAFs [10], pro-fibrotic stromal remodeling [11], and hypoxia-induced metabolic reprogramming [12]. Notably, PD-L1 is widely expressed on TNBC cells within the TME [13], where it binds PD-1 on T cells to suppress the activation and function of cytotoxic T lymphocytes, ultimately facilitating tumor immune evasion [14]. Consequently, pembrolizumab/atezolizumab (PD-1/PD-L1 inhibitors) combined with neoadjuvant chemotherapy was approved for advanced TNBC [15]. In the KEYNOTE-355 trial, pembrolizumab plus chemotherapy extended median progression-free survival (PFS) to 9.7 months vs. 5.6 months with chemotherapy alone (Hazard Ratio 0.65) [16]. Moreover, PD-L1 not only suppresses T-cell immunity but also activates MAPK (MEK-ERK) and PI3K-AKT pathways to promote chemoresistance [17].

Concurrently, chemokine CXCL12, the ligand for the G-protein-coupled receptor CXCR4, specifically interacts with its receptor and drives multiple pro-tumor processes through the CXCL12/CXCR4 axis, including promoting tumor cell proliferation, metastasis, immune evasion, and stromal remodeling [18,19]. CXCR4 is highly expressed in TNBC [20] and correlates with poor prognosis and distant metastasis [21]. Moreover, the CXCL12/CXCR4 axis upregulates matrix metalloproteinases (MMPs), which are key extracellular matrix remodeling factors [22], drives epithelial–mesenchymal transition [23], and maintains cancer stem cell (CSC) self-renewal [24]. CSCs function as chemoresistance reservoirs through ATP-binding cassette (ABC) transporter-mediated drug efflux and enhanced DNA repair [25].

Accumulating evidence indicates a mechanistic association between PD-L1 and CXCR4 in TNBC treatment and drug resistance [26,27]. By recruiting myeloid-derived suppressor cells (MDSCs), tumor-associated macrophages (TAMs), and Tregs, while simultaneously promoting stromal remodeling, CXCL12 physically hinders the effective infiltration of cytotoxic T lymphocytes into the tumor core, thereby establishing an immunosuppressive microenvironment that attenuates the efficacy of PD-L1 blockade [28]. This spatial segregation of immune effector cells from tumor cells, combined with local immunosuppression, results in an immune-excluded TME that is refractory to single-agent checkpoint blockade [29]. Therefore, suppression of CXCL12 abolishes the immune-privileged state by promoting T-cell infiltration into tumors, thereby complementing checkpoint inhibition and augmenting anti-PD-L1 immunotherapy [30]. Preclinical studies [31] have shown that CXCR4 blockade acts synergistically with anti-PD-1/PD-L1 checkpoint inhibitors to remodel the tumor stromal microenvironment and relieve immunosuppression, which not only curbs the accumulation of MDSCs, CAFs, and Tregs but also strengthens the cytotoxic function of CD8^+^ T cells. This dual-targeting strategy exerts complementary effects that overcome TME immunosuppression and drug resistance, emerging as a promising approach for TNBC therapy.

Compared with conventional IgG antibodies, nanobodies (Nbs), which are the variable domain of heavy-chain antibodies (VHH), are small in size with strong tissue penetration and cavity binding capabilities, allowing them to cross tissue barriers and accumulate in target organs or solid tumors such as breast cancer and pancreatic cancer [32]. Our prior study demonstrated that the bispecific nanobody BsNb PX4 (anti-PD-L1&CXCR4), generated through flexible linker-mediated fusion of single-domain antibody fragments, significantly suppressed subcutaneous pancreatic tumor growth in NOD/SCID mice [33]. By inhibiting CXCR12 (Stromal Cell-Derived Factor 1 Alpha, SDF-1α) secretion and alpha smooth muscle actin (α-SMA) expression in pancreatic stellate cells, BsNb PX4 impedes the activation of cancer-associated fibroblasts, which remodels the pancreatic tumor stromal microenvironment and enhances T-cell infiltration into tumor tissue [34].

In this study, we combined the dual-targeting molecule BsNb PX4 with chemotherapeutic agents to evaluate its antitumor efficacy in TNBC models both in vitro and in vivo. Our findings confirmed that the bispecific nanobody BsNb PX4 remodels the immunosuppressive microenvironment and enhances chemosensitivity, providing a solid rationale for addressing current therapeutic limitations in TNBC.

## 2. Results

### 2.1. Morphological and Size Characterization of BsNb PX4

**Figure 1:**
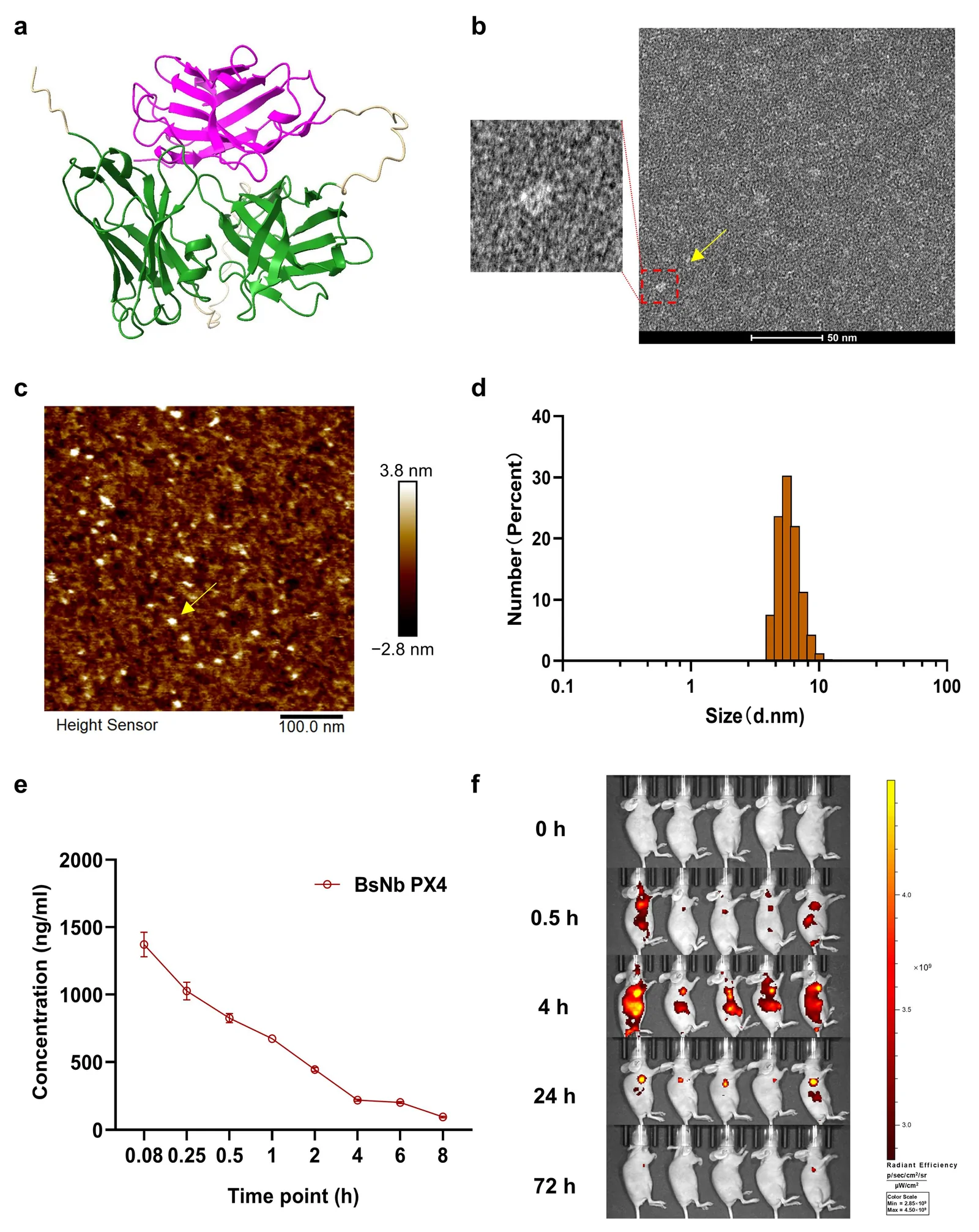
Characterization and in vivo behaviors of BsNb PX4. (**a**) Predicted three-dimensional structure of the trivalent bispecific nanobody generated by AlphaFold3. The construct is composed of one anti-PD-L1 VHH (purple) and two anti-CXCR4 VHHs (green) linked by flexible peptide linkers (wheat). (**b**) TEM micrograph of BsNb PX4. Yellow arrow indicates representative BsNb PX4 particle showing the typical morphology. (**c**) AFM analysis of BsNb PX4. Yellow arrow indicates representative BsNb PX4 particle. (**d**) Hydrodynamic diameter distribution of BsNb PX4 measured by DLS. (**e**) Pharmacokinetic fitting curves of BsNb PX4 (*n* = 5). (**f**) In vivo fluorescence imaging of Cy5.5-labeled BsNb PX4 (*n* = 5).

The structural morphology and size distribution of BsNb PX4 were first characterized. BsNb PX4 exhibited morphological features similar to the three-dimensional structure predicted by AlphaFold3 (Figure 1a), as revealed by transmission electron microscopy (TEM) (Figure 1b). Atomic force microscopy (AFM) analysis further showed an average particle size of approximately 6.67 nm (Figure 1c); consistently, dynamic light-scattering (DLS) analysis showed a mean hydrodynamic diameter of 6.585 nm with a polydispersity index (PDI) of 0.187 (Figure 1d), indicating that BsNb PX4 maintained a uniform nanoscale size distribution.

### 2.2. In Vivo Behaviors of BsNb PX4

The non-compartmental model was used to determine the pharmacokinetic parameters of BsNb PX4, which displayed a (t_1_/_2_) of 2.47 h (Figure 1e; Table S1), consistent with the characteristic rapid clearance profile of unmodified nanobodies.

The tumor-homing capacity and biodistribution of BsNb PX4 were investigated in MDA-MB-231 tumor-bearing BALB/c nude mice using real-time fluorescence imaging. As shown in Figure 1f, following intravenous injection of Cy5.5-labeled BsNb PX4, distinct fluorescence signal accumulation in the tumor region was observed from 0.5 h to 4 h; the signal accumulated predominantly at the tumor site by 24 h and persisted until 72 h post-injection. Immunohistochemistry staining for the His-tagged BsNb PX4 at 72 h post-administration exhibited a robust signal in tumor tissue, moderate signals in liver and kidney, and minimal signals in lung and spleen (Figure S1), consistent with in vivo imaging and confirming efficient tumor accumulation and prolonged retention.

### 2.3. High Expression of PD-L1 and CXCR4 Was Positively Correlated in TNBC and Chemotherapy Treatment Upregulated PD-L1 and CXCR4 Levels

**Figure 2:**
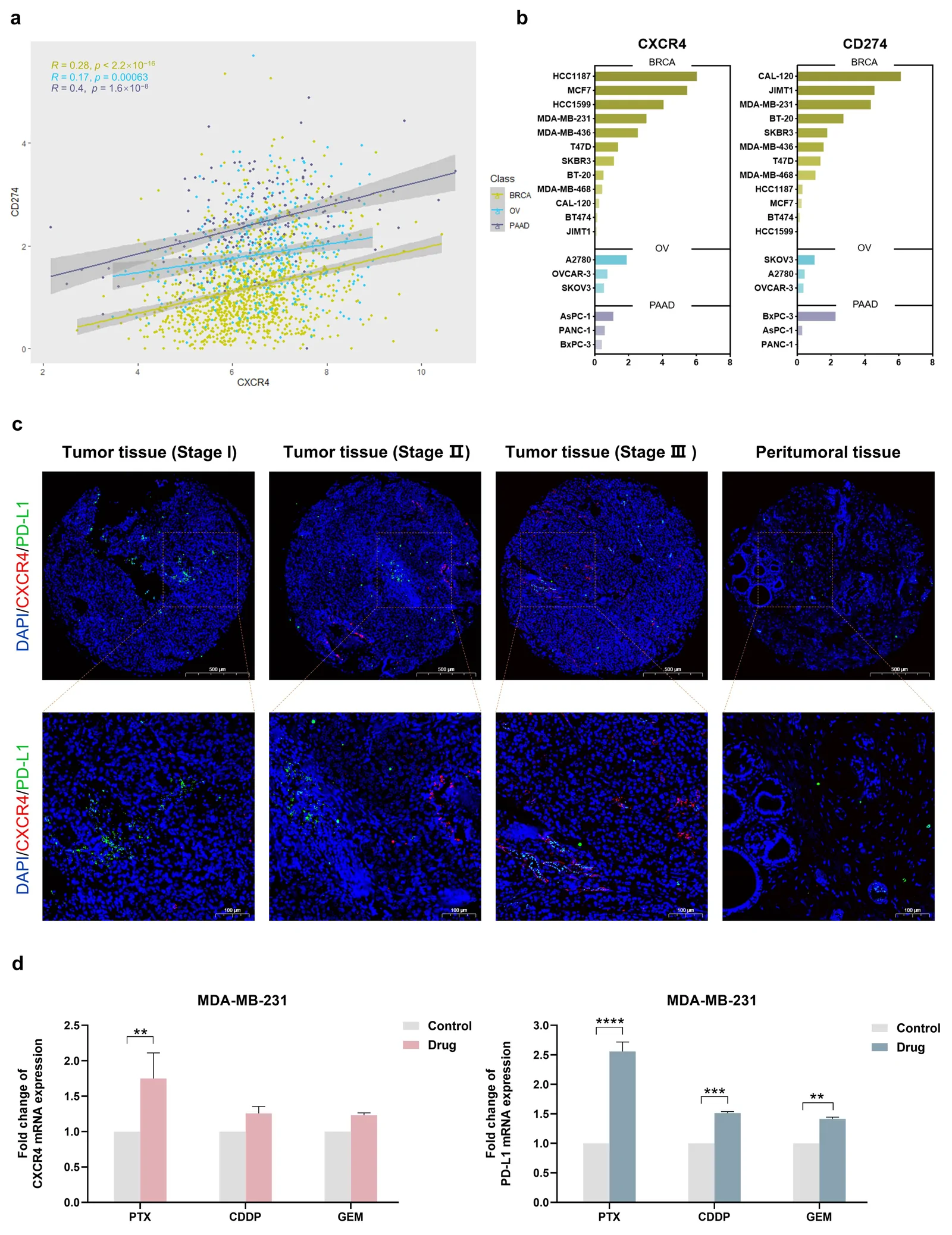
Correlation between PD-L1 and CXCR4 expression and their chemotherapy-induced upregulation in breast cancer cells. (**a**) Linear curve fitted between PD-L1 (CD274) and CXCR4 levels based on TCGA database (breast cancer, BRCA, *n* = 1104; ovarian cancer, OV, *n* = 379; pancreatic adenocarcinoma, PAAD, *n* = 182). (**b**) Gene expression levels of PD-L1 and CXCR4 in different tumor cell types. Colors indicate cancer types: yellow, BRCA; blue, OV; purple, PAAD. (**c**) Immunofluorescence detection of CXCR4 and PD-L1 expression in TNBC clinical samples. (**d**) qPCR determined the transcription levels of PD-L1 and CXCR4 after chemotherapeutic drug treatment. All data are normalized to β-actin and presented as fold change (*n* = 3). Significance determined by one-way ANOVA with Tukey’s test. **** *p* < 0.0001; *** *p* < 0.001; ** *p* < 0.01.

Given the correlation mechanism between PD-L1 and CXCR4 in TNBC, the relationship between their expression levels was first examined at the transcriptomic level using The Cancer Genome Atlas (TCGA) database (Figure 2a), revealing a positive correlation in breast cancer (R = 0.28; *p* < 2.2 × 10^−16^). According to the Cancer Cell Line Encyclopedia (CCLE), gene expression levels of PD-L1 and CXCR4 vary across cancer cell lines, with several TNBC lines such as MDA-MB-231 exhibiting high co-expression of both markers (Figure 2b). Immunofluorescence staining of a TNBC tissue microarray revealed obvious co-expression of PD-L1 and CXCR4 in stage I–III tumor tissues, whereas only minimal PD-L1 expression and no CXCR4 signal were observed in peritumoral tissues (Figure 2c). Then, we examined surface expression of PD-L1 and CXCR4 on tumor cells by flow cytometry, as illustrated in Figure S2. Upon treatment with paclitaxel or gemcitabine, MDA-MB-231 cells demonstrated a marked increase in surface expression of both PD-L1 and CXCR4, while AsPC-1 cells showed a moderate upregulation of these molecules. Paclitaxel (PTX) or gemcitabine (GEM) likely caused stronger upregulation of these markers on the surface of tumor cells than cisplatin (CDDP). To determine whether the increased surface expression corresponded to transcriptional upregulation, we quantified PD-L1 and CXCR4 mRNA levels (normalized to β-actin as a housekeeping gene) in tumor cells after drug treatment, and found that low-dose chemotherapeutic drugs consistently upregulated the mRNA levels of both molecules in MDA-MB-231 cells (Figure 2d). Our data were consistent with findings from a clinical analysis, which revealed that bulk stranded RNA sequencing of primary breast cancer patients who underwent neoadjuvant chemotherapy detected significantly higher CXCR4 expression after treatment than before (*p* = 0.018, Figure S3).

### 2.4. BsNb PX4 Enhanced Sensitivity of Chemotherapy In Vitro

**Figure 3:**
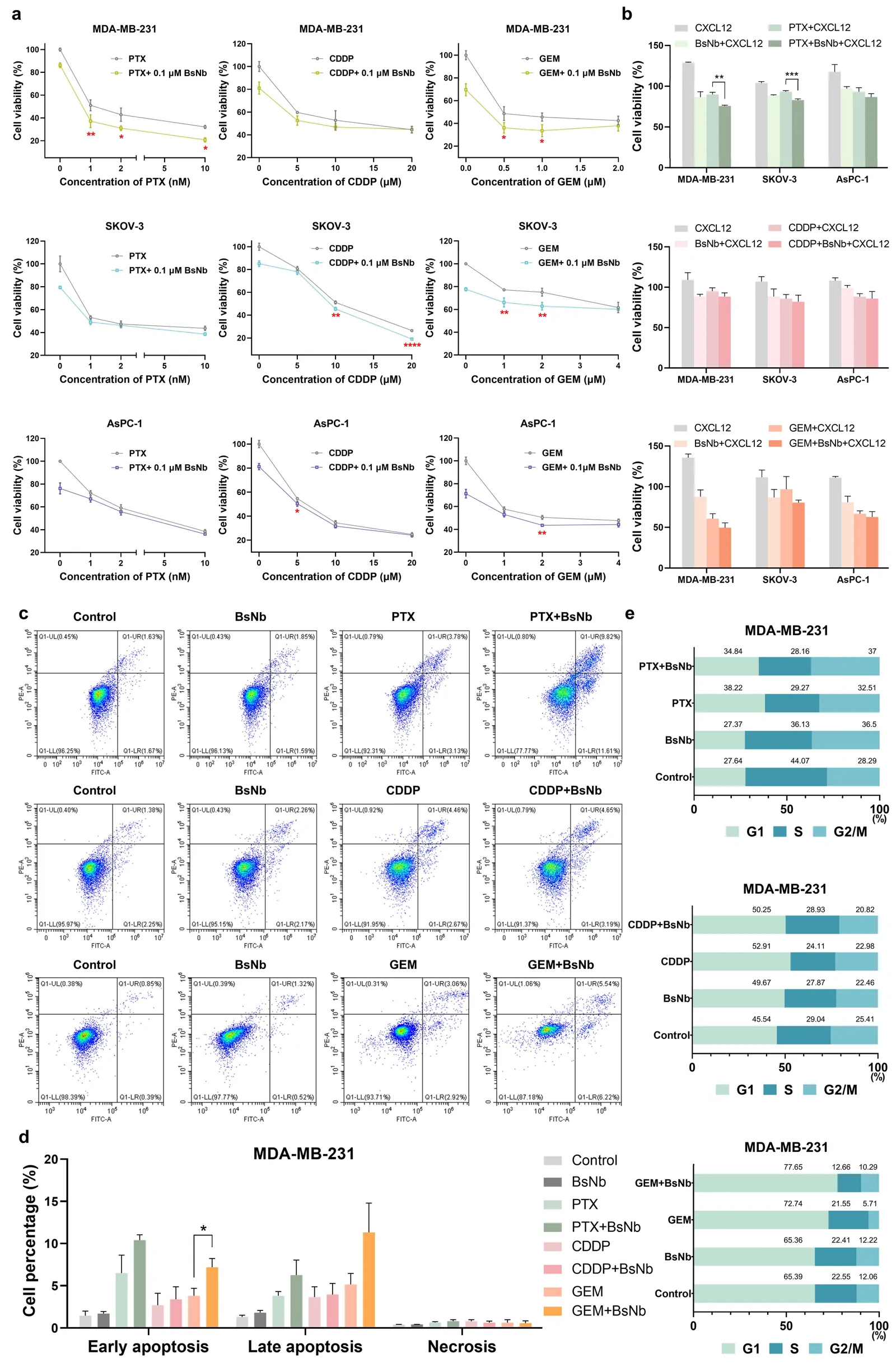
In vitro analysis of cell viability, apoptosis, and cell cycle distribution. (**a**) The viability of tumor cells treated with BsNb PX4 or a chemotherapeutic drug, alone or in combination. Cell viability was detected through the CCK-8 kit. The asterisk denotes a statistically significant difference between the combination group and the corresponding monotherapy group at the same chemotherapeutic drug concentration (*n* = 3). (**b**) Inhibition of tumor cell proliferation resulted from a bispecific nanobody combined with a chemotherapeutic drug in the presence of 200 ng/mL CXCL12 (*n* = 3). (**c**) Comparison of tumor cell apoptosis rate induced by different drug combinations. MDA-MB-231 cells were incubated with 0.1 µM BsNb PX4 and/or 1 nM PTX, 2 μM GEM, or 5 μM CDDP, respectively (*n* = 3). The cells were collected and subjected to analysis by flow cytometry. (**d**) The histogram of early and late apoptotic cells. Data are summarized as the mean ± SD of three independent experiments. Significance was determined by one-way ANOVA with Tukey’s test. **** *p* < 0.0001; *** *p* < 0.001; ** *p* < 0.01; * *p* < 0.05. (**e**) Cell cycle assay of MDA-MB-231 cells by flow cytometry. Data was analyzed by Flowjo.V10 (BD Biosciences).

To assess whether BsNb PX4 could sensitize TNBC cells to chemotherapy, paclitaxel (PTX), gemcitabine (GEM), and cisplatin (CDDP) were selected as representative chemotherapeutic agents commonly used in TNBC treatment, with three concentrations close to the IC50 of each drug chosen based on dose–response curves (Figure S4; Table S2). BsNb PX4 combined with low-dose PTX or GEM significantly reduced MDA-MB-231 cell viability compared with monotherapy (Figure 3a). Notably, the combination of BsNb PX4 with CDDP also reduced cell viability more in SKOV3 and AsPC-1 cells compared with CDDP alone, whereas no significant reduction was observed in MDA-MB-231 cells. As shown in Figure 3b, CXCL12 promoted the proliferation of tumor cells, and the combination of BsNb PX4 with chemotherapeutic drugs significantly inhibited MDA-MB-231 cell growth compared to PTX monotherapy. These findings demonstrate that the bispecific nanobody BsNb PX4 enhances the antitumor efficacy of PTX by blocking the CXCL12/CXCR4 signaling pathway.

We also evaluated the effects of BsNb PX4 and the chemotherapeutic drug, alone and in combination, on apoptosis using annexin V-FITC/PI detection. Treatment with 1 nM PTX induced substantial apoptosis, 2 μM GEM resulted in a moderate apoptotic rate (Figure 3c,d), and cisplatin exhibited the weakest apoptosis-promoting effect in MDA-MB-231 cells. The combination of BsNb PX4 with PTX or GEM significantly increased the early apoptosis rate compared to each monotherapy (10.40 ± 0.63% vs. 6.49 ± 2.14%; 7.19 ± 1.04% vs. 3.80 ± 0.88%, *p* < 0.05). As illustrated in Figure 3e, the combination treatment of BsNb PX4 and low-dose PTX (1 nM) further reduced the S-phase proportion of MDA-MB-231 cells, while increasing the G2/M-phase proportion (37% vs. 32.51% in the PTX monotherapy group). This synergistic interaction results in cell cycle arrest and subsequent apoptosis, thereby effectively suppressing tumor cell proliferation, and ultimately appears to potentiate the cellular sensitivity to PTX.

### 2.5. BsNb PX4 Restored the Cytotoxicity of hPBMCs in Chemotherapy

**Figure 4:**
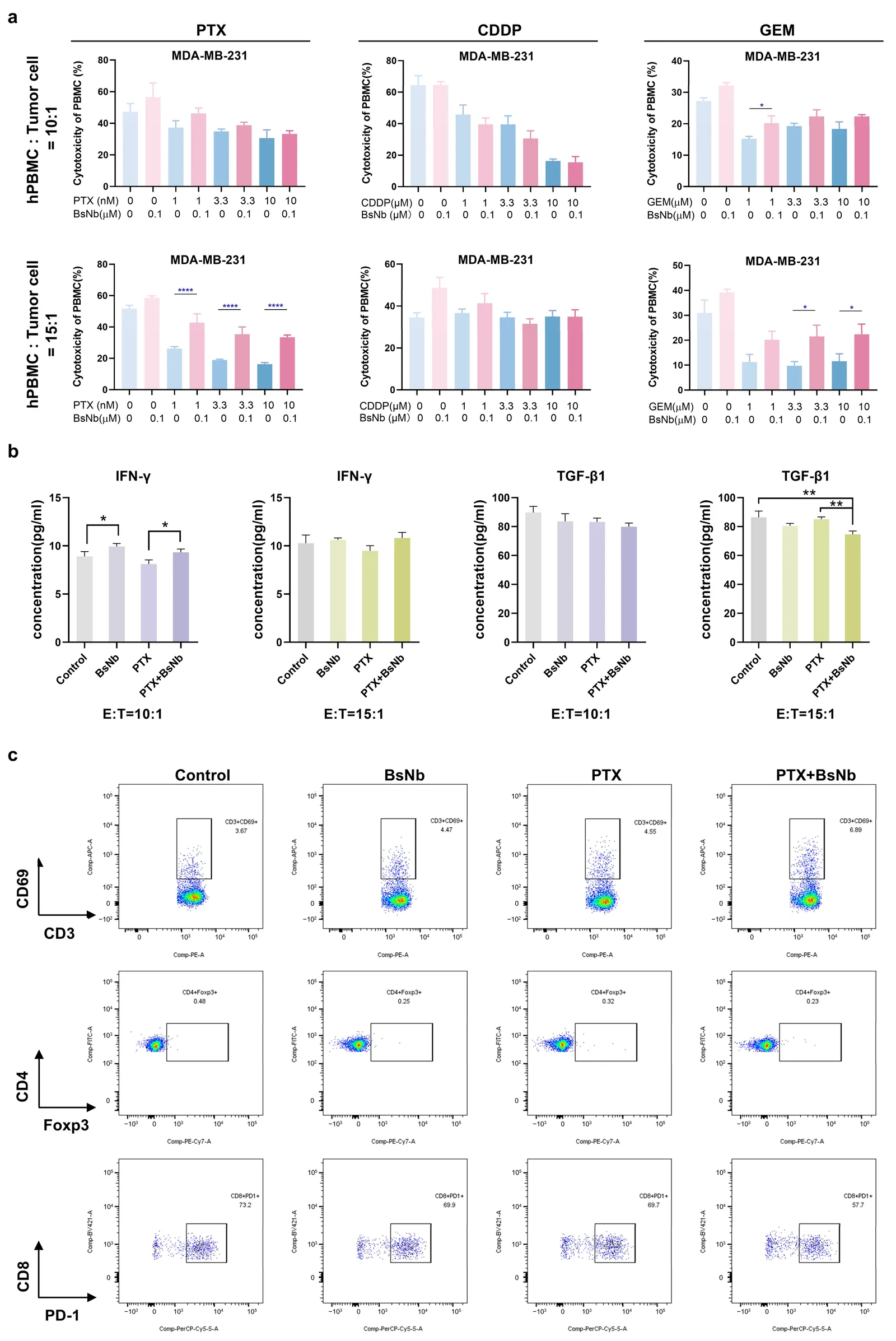
The combination of paclitaxel and BsNb PX4 remodeled the tumor immune microenvironment in vitro. (**a**) The effect of a chemotherapy drug with BsNb PX4 on the cytotoxicity of hPBMCs in vitro (hPBMC: tumor cell = 10:1; hPBMC: tumor cell = 15:1; *n* = 3). (**b**) The effect of BsNb PX4 combined with paclitaxel (1 nM) on the release of cytokines in a hPBMC and tumor cell co-incubation system (*n* = 3). (**c**) Analysis of T-lymphocyte subgroups in co-cultures of MDA-MB-231 with hPBMCs, with different treatment groups. Data are representative of three independent experiments. Significance determined by one-way ANOVA with Tukey’s test. **** *p* < 0.0001; ** *p* < 0.01; * *p* < 0.05.

To determine whether BsNb PX4 could restore the cytotoxic function of human PBMCs (hPBMCs) against MDA-MB-231 cells in the presence of chemotherapy, hPBMCs and tumor cells were co-cultured at E:T ratios of 10:1 and 15:1, and cell lysis was quantified by the LDH release assay. As shown in Figure 4a, at an E:T ratio of 15:1, the combination of PTX and BsNb PX4 significantly restored hPBMC-mediated killing activity compared to PTX monotherapy (*p* < 0.0001).

As illustrated in Figure 4b, BsNb PX4 monotherapy significantly increased IFN-γ secretion in hPBMC–tumor cell co-cultures compared to controls (*p* < 0.05) at an E:T ratio of 10:1, indicating enhanced immune activation and tumoricidal activity. The combination of BsNb PX4 and PTX (1 nM) synergistically amplified IFN-γ production (*p* < 0.05, vs. PTX alone) (Figure 4b), likely attributable to PTX-induced immunogenic cell death (ICD), which may release damage-associated molecular patterns (DAMPs) to boost DC-mediated antigens presentation [35]. Importantly, combined therapy markedly reduced TGF-β1 levels compared to control and monotherapy groups (*p* < 0.01) (Figure 4b). IL-6 activates the JAK/STAT3 pathway in the tumor microenvironment, where STAT3 initiates the transcription of Cyclin D1 and HIF-1α, thereby contributing to resistance against chemotherapy-induced cytotoxicity and apoptosis [36,37], while promoting tumor cell proliferation and EMT-mediated invasion [38]. Moreover, low-dose PTX (1 nM) slightly increased the pro-inflammatory cytokine IL-6’s secretion, while the combination treatment resulted in a lower level than PTX monotherapy (Figure S5).

Further analysis of T-cell subsets indicated a higher proportion of CD69^+^ cells among CD3^+^ T cells in the PTX + BsNb PX4 group than in the other groups, reaching 6.89% (Figure 4c). Additionally, we analyzed the expression of the regulatory T-cell (Treg) marker Foxp3, and flow cytometry results demonstrated that tumor cells treated with BsNb alone or in combination induced a considerable decrease in its expression compared with other groups, particularly the control. Notably, the proportion of PD-1^+^ CD8^+^ T cells was reduced in the combination treatment compared with PTX alone (57.7% vs. 69.7%), suggesting attenuated CD8^+^ T-cell exhaustion.

### 2.6. The Combination of PTX and BsNb PX4 Treatment Exerts Synergistic Antitumor Effects in TNBC Xenograft Mouse Model

**Figure 5:**
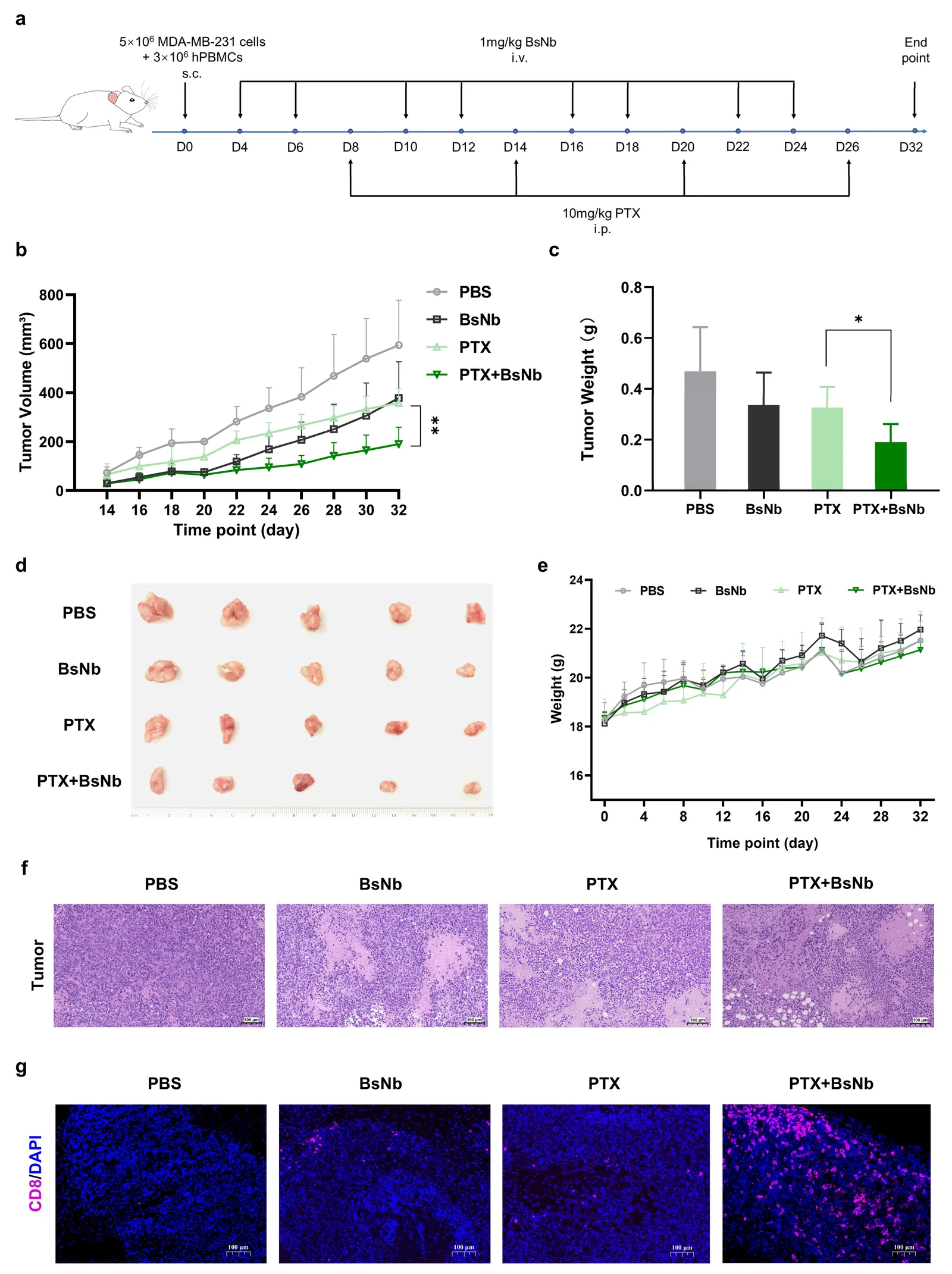
BsNb PX4 combined with PTX enhanced the antitumor effect of the MDA-MB-231/hPBMC co-grafting model (*n* = 5). (**a**) Schematic schedule of inoculation and treatment. Mice had been randomized based on body weight on the day of inoculation. (**b**) Tumor volume change curves of different treatment groups. Tumor volumes were first measured on Day 14, and the statistical significance of tumor volumes at the end point was calculated using two-way ANOVA with Tukey’s test (** *p* < 0.01). (**c**) The weight of the tumor at the end of the experiment. Significance was determined by an unpaired *t*-test (* *p* < 0.05). (**d**) All tumor masses were photographed. (**e**) Weight change curve of mice in different treatment groups. (**f**) HE staining of tumor tissues in the MDA-MB-231/hPBMC co-grafting model. (**g**) Immunofluorescence staining of tumor tissues in different treatment groups.

The antitumor efficacy of BsNb PX4 combined with PTX was further evaluated in an hPBMC co-transplantation model. Compared with PBS controls, both the PTX and BsNb PX4 monotherapy groups showed tumor growth inhibition (Figure 5b). Strikingly, the combination group achieved maximal tumor suppression as well as minimal tumor weight, showing statistically significant differences in final tumor volume (*p* < 0.01, two-way ANOVA with Tukey’s post hoc test) compared to the PTX monotherapy group, consistent with in vitro findings (Figure 5b–d). Hematoxylin–eosin (HE) staining of the tumor sections revealed partial necrotic areas in both the PTX and BsNb PX4 monotherapy groups compared to PBS controls, with BsNb PX4 specimens displaying characteristic vacuolar structures. The combination group exhibited expanded necrotic regions and vacuolization, indicating synergistic antitumor activity (Figure 5f). Immunofluorescence analysis of CD8^+^ T-cell infiltration showed minimal recruitment in PBS groups, mild infiltration with PTX or BsNb PX4 monotherapy, and robust CD8^+^ T-cell accumulation in combination-treated tumors (Figure 5g). These findings suggest that BsNb PX4 facilitates both CD8^+^ T-cell recruitment and chemosensitization to PTX, potentially through enhanced drug penetration via tumor microenvironment modulation.

**Figure 6:**
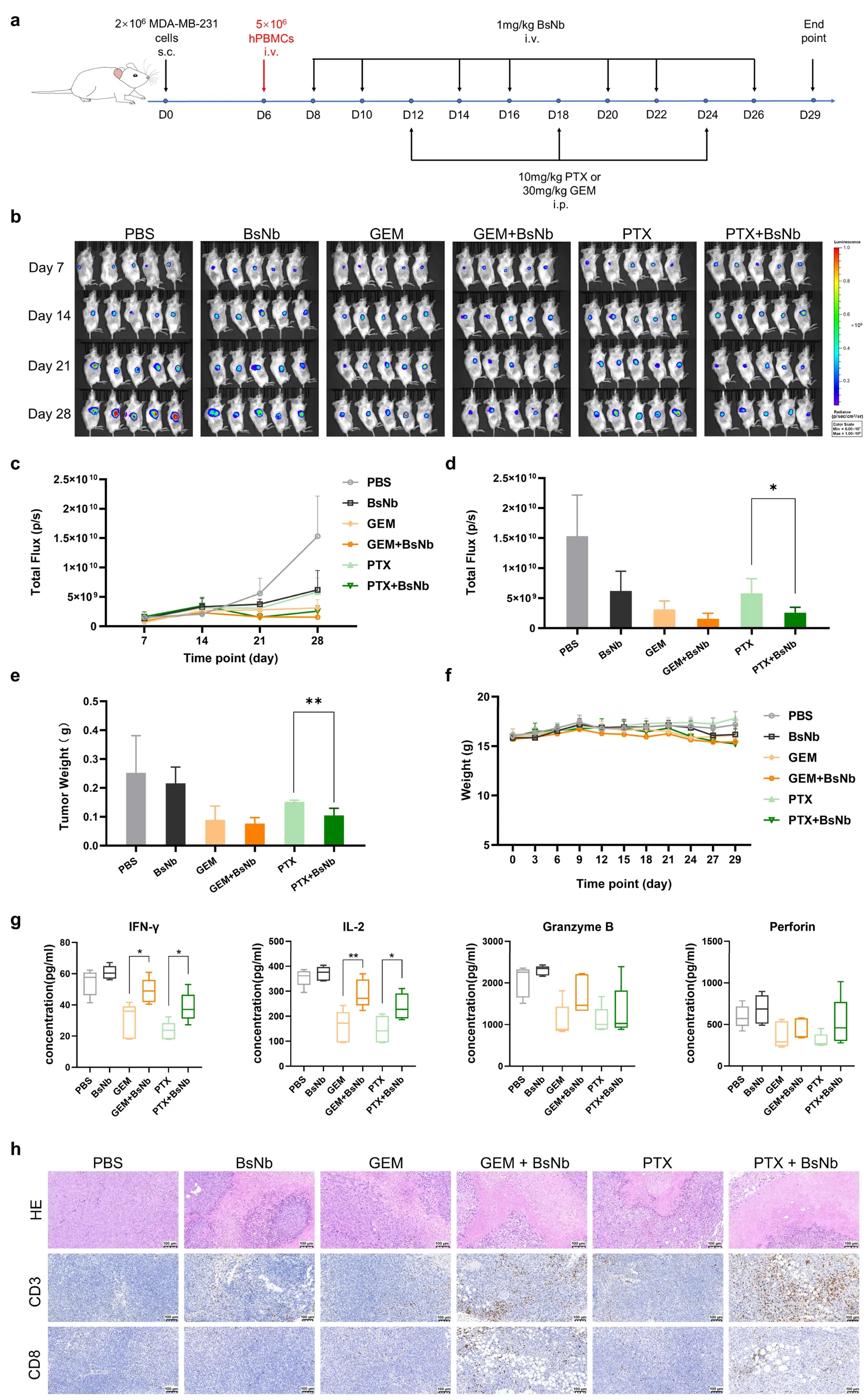
BsNb PX4 combined with chemotherapeutic drugs enhances the antitumor effect of the MDA-MB-231-luc xenograft model with intravenous transfer of hPBMCs (*n* = 5). (**a**) Schematic schedule of inoculation and treatment. (**b**) Fluorescence imaging of MDA-MB-231-luc tumors in NOG mice in different treatment groups. (**c**) Tumor fluorescence intensity during treatment. (**d**) Tumor fluorescence intensity at the end of the experiment. (**e**) The weight of the tumor at the end of the experiment. (**f**) Weight change curve of mice in different treatment groups. (**g**) The concentration of cytokines in mice serum, including IFN-γ, IL-2, Granzyme B, and Perforin. (**h**) HE and immunohistochemistry staining of tumor tissues. Significance determined by one-way ANOVA with Tukey’s test. ** *p* < 0.01; * *p* < 0.05.

To monitor tumor progression distinctly, MDA-MB-231-luc cells were subcutaneously inoculated into NOG mice with intraperitoneal transfer of hPBMCs. As shown in Figure 6b, weekly in vivo bioluminescence imaging was performed to quantify tumor growth with ROI (region-of-interest) analysis (total flux, p/sec/cm^2^/sr). All treatment groups showed reduced fluorescence intensity compared to PBS controls (Figure 6c,d); importantly, PTX + BsNb PX4 combination therapy demonstrated statistically superior tumor volume and weight (*p* < 0.01, unpaired *t*-tests) reduction vs. PTX monotherapy, underscoring the enhanced immunotherapeutic synergy with chemotherapy (Figure S7a,b and Figure 6e). Correlating with the antitumor efficacy, the combination therapy significantly enhanced secretion of cytotoxic effector molecules (IFN-γ, IL-2) compared with chemotherapy alone, and showed a trend toward reduced IL-6 and TGF-β1 levels. (Figure 6g and Figure S7c). CD3 and CD8 staining results indicated maximum T-cell infiltration in tumors from the PTX + BsNb PX4 and GEM + BsNb PX4 groups, suggesting that the combination therapy effectively promoted the recruitment and activation of cytotoxic T cells within the tumor microenvironment, leading to robust T-cell-mediated antitumor activities (Figure 6h).

These results preliminarily demonstrated that BsNb PX4 enhanced sensitivity to chemotherapy in PD-L1- and CXCR4-positive TNBC xenograft models through enhancing the immune responses.

### 2.7. In Vivo Safety Evaluation of the Combination Therapy

The systemic safety of the combination therapy was assessed in both xenograft models. In the MDA-MB-231/hPBMC xenograft model, all treatment groups exhibited progressive weight gain throughout the study, with no statistically significant intergroup differences in terminal body weights, indicating negligible systemic toxicity (Figure 5e). As demonstrated in Figure S6a, serum ALT, AST, CREA-S, and UA levels remained within normal murine physiological ranges across all groups. TP II levels were reduced in both the PTX and PTX + BsNb PX4 groups, potentially attributable to PTX-induced renal tubular epithelial injury affecting protein reabsorption, as suggested by HE staining (Figure S6b). Concurrently, the elevated urea concentration may arise from renal functional alterations and/or increased protein catabolism.

In the MDA-MB-231-luc/hPBMC sequential tail vein inoculation model, body weights increased mid-treatment but declined terminally, probably correlating with graft-versus-host disease (GVHD) progression (Figure 6f). Peripheral blood analysis revealed normal WBC and HGB levels across groups (Figure S7d). GEM monotherapy induced leukopenia (WBC: 6.49 ± 2.92 × 10^9^/L vs. 13.89 ± 6.64 × 10^9^/L in controls) and thrombocytopenia (PLT: 431.2 ± 335.5 × 10^9^/L vs. 973.4 ± 316.1 × 10^9^/L in controls), consistent with clinical chemotoxicity profiles; co-administration of BsNb PX4 partially mitigated these effects (WBC: 8.74 ± 5.37 × 10^9^/L; PLT: 780.6 ± 356.7 × 10^9^/L). Although the combination group showed significant alterations in RBC levels compared to controls (*p* = 0.041), no marked histopathological damage was observed in major organs (Figure S7e).

These findings demonstrate that the bispecific nanobody BsNb PX4 combined with PTX has preliminarily favorable safety in MDA-MB-231/hPBMC xenograft models.

### 2.8. BsNb PX4 and Paclitaxel Combination Modulates Tumor Microenvironment In Vivo

**Figure 7:**
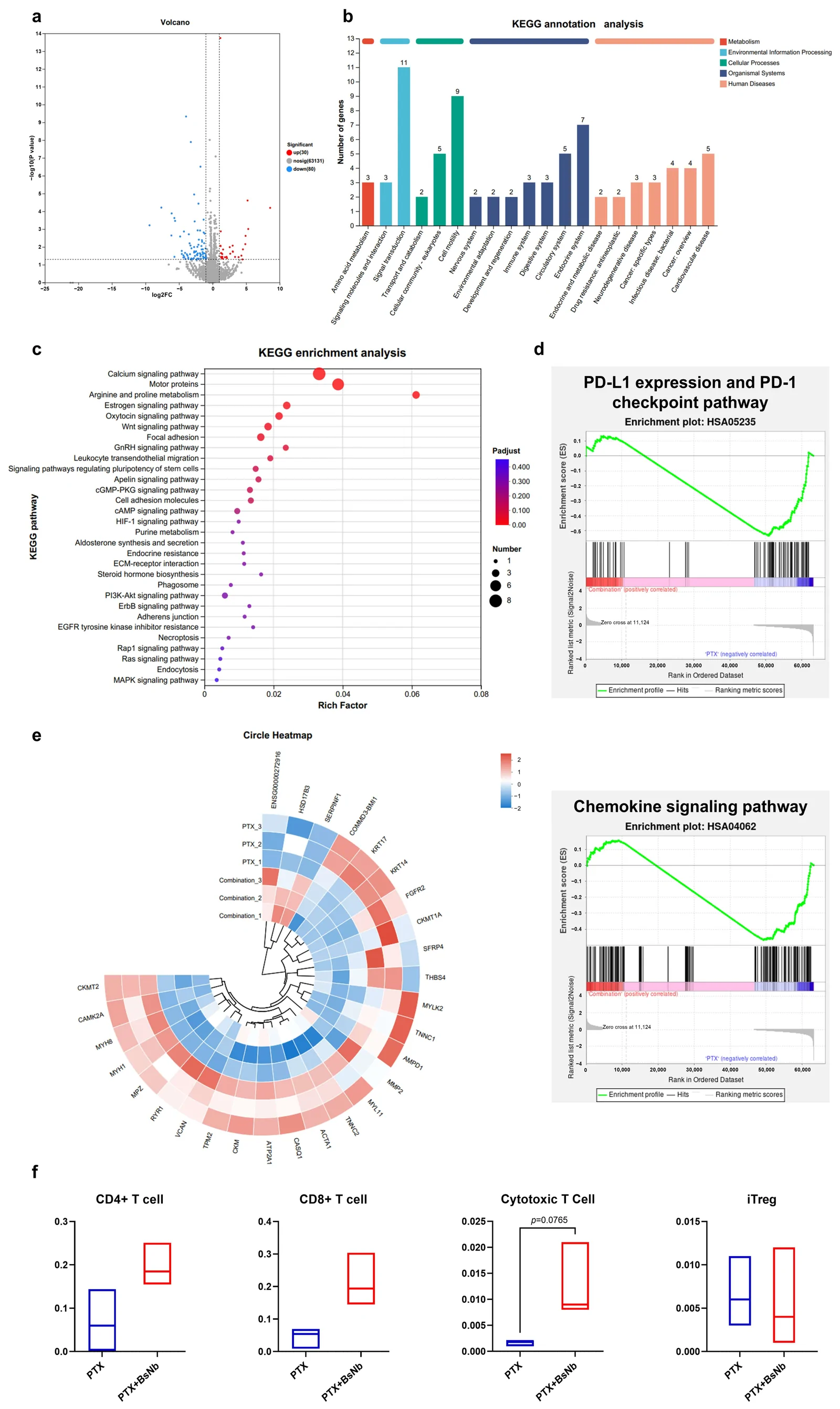
Transcriptomic analyses of the MDA-MB-231/hPBMC co-grafting model after PTX and BsNb PX4 combination treatment. (**a**) Volcano plot of the differentially expressed genes between the PTX and combination treatment (PTX + BsNb) groups (*n* = 3). Blue and red spots represent down- and upregulated genes (*p*-value < 0.05 and log2 (FC) ˃ 2). (**b**) KEGG annotation analysis of the differentially expressed genes. (**c**) KEGG pathway enrichment analysis of the differentially expressed genes. (**d**) GSEA of the gene set in PD-L1 expression and the PD-1 checkpoint pathway. The normalized enrichment score (NES) was −1.15, and *p*-adjust was 0.20. GSEA of the gene set in the chemokine signaling pathway. The NES was −1.08, and *p*-adjust was 0.28. Each black line represents a gene in the set; the red-blue heatmap shows expression ranking, where deeper red indicates higher log2 (FC) and deeper blue indicates lower log2 (FC). (**e**) Circle heatmap analysis of the genes implicated in the TME modulation. (**f**) Immune cell infiltration online analysis by ImmuCellAI. In the box-plots, blue represents the PTX monotherapy group, and red represents the PTX + BsNb PX4 combination group. Data were analyzed by unpaired *t*-tests and are shown as mean ± SD (*n* = 3).

To further investigate the mechanism of the combination treatment, the total RNA of stripped tumors in the PTX and combination (PTX + BsNb PX4) groups was extracted and analyzed using RNA sequencing. A total of 110 significant DEGs (differentially expressed genes) were filtered using the criteria of a *p*-value of 0.05 and log2 (fold change, FC) of 2, including 30 upregulated and 80 downregulated genes (Figure 7a). Additionally, Kyoto Encyclopedia of Genes and Genomes (KEGG) annotation analysis showed that the DEGs are associated with signal transduction, cell motility, the endocrine system, and drug resistance (Figure 7b). KEGG enrichment analysis further revealed the antitumor mechanisms of the combination treatment: it modulates multiple pathways, including the calcium, MAPK, Wnt, and PI3K-AKT signaling pathways; the arginine and proline metabolism pathway; leukocyte transendothelial migration, and ECM-receptor interaction (Figure 7c). The DEGs were presented in the circle heatmap based on the KEGG enrichment analysis (Figure 7e). Gene Set Enrichment Analysis (GSEA) indicated that the combination treatment could negatively regulate PD-L1 expression and the PD-1 checkpoint pathway as well as the chemokine signaling pathway (Figure 7d). To further evaluate immune cell infiltration in both groups, ImmuCellAI analysis was conducted. The combination treatment upregulated the immune infiltration degree associated with higher cytotoxic T-cell and lower inducible regulatory T (iTreg)-cell levels (Figure 7f).

## 3. Discussion

Frequent emergence of chemoresistance acts as the principal trigger for therapeutic failure and disease recurrence in TNBC [39]. An increasing body of evidence indicates that the tumor microenvironment, a complex system comprising immune cells, stromal components, and a milieu of signaling molecules, is a significant player in altering drug response and enabling resistance mechanisms [10]. TNBC is more likely than other breast cancer subtypes to benefit from immune checkpoint blockade therapy due to its higher immunogenicity, enrichment by tumor-infiltrating lymphocytes (TILs), and levels of PD-L1 expression. This clinical benefit has been demonstrated in the phase 3 TORCHLIGHT trial, where toripalimab (anti-PD-1) plus nab-paclitaxel significantly prolonged progression-free and overall survival in patients with metastatic or recurrent TNBC [40]. Recent studies have shown that the efficacy of immune checkpoint inhibitors (ICIs) depends on the localization of immune cells, particularly T cells, within the TME.

Cancer-associated fibroblasts are key components of the TME that modulate T-cell immunity by secreting cytokines and forming structural barriers. CXCL12 secreted by CAFs engages CXCR4 on T cells to induce chemotaxis, while TGF-β, another humoral factor secreted by CAFs and tumor cells, has been reported to regulate the CXCL12/CXCR4 axis. A growing body of evidence further demonstrates that the CXCL12/CXCR4 axis induces epithelial–mesenchymal transition in tumor cells and promotes angiogenesis, thereby facilitating tumor growth and metastasis [41]. Previous studies have reported that TGF-β1 increases CXCR4 expression on human CD4^+^ T cells [42]. CAFs derived from human mammary carcinoma exhibit autocrine TGF-β signaling that upregulates CXCL12 expression via Smad2/3 activation, thereby leading to T-cell entrapment in the tumor stroma [43]. CXCL12 silencing significantly reduced SOX18-triggered TAM and Treg accumulation, while the combination of the TGF-β R1 inhibitor vactosertib or CXCR4 inhibitor with anti-PD-L1 potently blocked Hepatocellular Carcinoma (HCC) progression and metastasis [44]. Moreover, agents targeting TGF-β and PD-L1 exert potent antitumor effects by reducing collagen deposition and promoting CD8^+^ T-cell infiltration, thereby remodeling the tumor microenvironment.

Based on this rationale, we engineered BsNb PX4, a trivalent bispecific nanobody comprising one anti-PD-L1 VHH and two anti-CXCR4 VHHs connected by flexible linkers. Structurally, this design yielded a uniform nanoparticle with an average diameter of approximately 6.6 nm, which is desirable for tumor penetration. BsNb PX4 binds specifically to PD-L1 and CXCR4 on tumor and stromal cells, and we previously indicated the impact of blocking the CXCL12/CXCR4 and PD-1/PD-L1 signaling pathways on extracellular matrix deposition, which retards the epithelial–mesenchymal transition of pancreatic cancer cells and impedes the activation of cancer-associated fibroblasts, in turn facilitating the infiltration of T cells into tumor lesions. However, the biological effects of BsNb PX4 combined with chemotherapeutics on the tumor microenvironment still require further investigation.

In preclinical models, CXCR4 was expressed more frequently in TNBC than in other breast cancer subtypes, and its inhibition has been shown to re-sensitize TNBC cells to conventional chemotherapeutics, including doxorubicin, paclitaxel, and cisplatin, through the reversal of drug resistance mechanisms [45]. Our results showed that the combination of BsNb PX4 and PTX produced synergistic effects on chemosensitivity across MDA-MB-231, SKOV-3, and AsPC-1 cell lines, with efficacy varying among different cell types. Furthermore, BsNb PX4 could recover the cytotoxic function of hPBMCs against MDA-MB-231 cells in the presence of low-dose PTX. Although high-dose cytotoxic chemotherapy may suppress immunity via lymphocyte depletion [46], appropriately dosed regimens paradoxically enhance tumor-directed immune responses [47]. Intriguingly, increased PD-L1 expression was detected in tumor cells when co-cultured with hPBMCs or chemotherapeutic agents, and subsequent PD-1/PD-L1 blockade further restored antitumor cytotoxicity. Furthermore, this combination reshaped the tumor immune landscape by expanding CD8^+^ effector T cells and decreasing CD4^+^ Foxp3^+^ Tregs. These findings support the concept that CXCR4 blockade boosts immune infiltration within the tumor microenvironment by reducing immunosuppressive cells, thereby potentiating the efficacy of PD-1 inhibitors [27]. Furthermore, in the in vitro co-culture system, we also found that BsNb PX4 enhanced IFN-γ secretion and decreased TGF-β1 levels, suggesting that the immunomodulatory effects of BsNb PX4 are mediated through multiple complementary mechanisms. Notably, PTX activates TLR4-dependent NF-κB signaling in MDA-MB-231 cells, inducing autocrine IL-6 via this pathway [48], which contributes to chemotherapy-related side effects [49] and drug resistance [37].

The in vivo therapeutic efficacy of the combination was validated in the MDA-MB-231/hPBMC co-implantation model, which establishes a T-cell-enriched tumor microenvironment. However, TNBC and other clinical cold tumors are characterized by poor T-cell infiltration within an immunosuppressive TME, leading to reduced responses to immune checkpoint inhibitors. To better mimic clinical settings, we constructed an MDA-MB-231-luc with sequential hPBMC infusion model to reconstitute a humanized immune microenvironment. Consistently, the results revealed that the combined therapy achieved superior antitumor activity compared with single-agent chemotherapy, suggesting that BsNb PX4 potentiates T-cell-mediated cytotoxicity against MDA-MB-231 cells and normalizes the immunosuppressive TME, potentially facilitating the penetration of chemotherapeutic drugs.

To further explore the molecular basis, RNA-seq analysis identified significant alterations in multiple pathways in the combination group (vs. PTX monotherapy), which likely contribute to enhanced antitumor efficacy and increased paclitaxel sensitivity by regulating key mechanisms such as calcium signaling and MAPK pathways to modulate cell cycle progression, proliferation, and apoptosis. Mechanistically, the CXCL12/CXCR4 axis promotes TNBC invasion and metastasis via activation of the PI3K/AKT [50] and mTOR [51] pathways, and reciprocally regulates PD-L1 expression through PI3K/AKT/mTOR signaling, thereby promoting chemoresistance, CSC properties, and epithelial–mesenchymal transition [17]. This supports the concept that dual CXCR4/PD-L1 blockade is required to reverse TME immunosuppression [52]. Additionally, inhibition of the Wnt pathway reduced drug resistance [53], and modulation of immune-related pathways (e.g., leukocyte transendothelial migration) may enhance immune cell recruitment [54], consistent with the success of other combinatorial targeted therapies in TNBC.

Despite these promising findings, the complexity and heterogeneity of the tumor microenvironment in the clinical setting may substantially influence therapeutic efficacy and safety profiles. The precise molecular mechanisms governing the synergistic interaction between BsNb PX4 and chemotherapeutic agents, particularly the temporal dynamics of immune cell remodeling and stromal modulation, remain to be fully elucidated. Future studies should focus on identifying predictive biomarkers for patient stratification and conducting long-term toxicity assessments.

## 4. Materials and Methods

### 4.1. Cell Lines and Cell Culture

AsPC-1, SKOV3, and MDA-MB-231 cell lines were purchased from the Cell Bank of the Chinese Academy of Sciences (Shanghai, China). AsPC-1 cells were maintained in RPMI-1640 (Gibco, Grand Island, NY, USA), SKOV-3 cells were maintained in McCoy’s 5A (Gibco), and MDA-MB-231 cells were maintained in DMEM (Gibco), all of which were supplemented with 10% FBS (Gibco). MDA-MB-231-Luc cells were generated as described previously [55], and human peripheral blood mononuclear cells (hPBMCs) were obtained from SailyBio (Shanghai, China).

### 4.2. Characterization of BsNb PX4

An atomic force microscope (AFM, Bruker, Dimension FastScan Bio, Billerica, MA, USA) and a transmission electron microscope (TEM, Talos F200C G2, Thermo Fisher Scientific, Waltham, MA, USA) were used to observe the size and morphology of BsNb PX4. Dynamic light scattering (DLS, Zetasizer Nano ZS90, Malvern, UK) was utilized to determine the hydrodynamic diameter and polydispersity index.

### 4.3. Acquisition and Bioinformatic Analysis of Cell Line and Clinical Datasets

PD-L1 and CXCR4 gene expression levels in human cancer cell lines were downloaded from the Cancer Cell Line Encyclopedia (https://portals.broadinstitute.org/ccle, accessed on 23 June 2026). TCGA data elaborating on the relevance of expression were downloaded from Xena (https://xenabrowser.net/datapages/, accessed on 23 June 2026) to assess the pairwise correlation between PD-L1 and CXCR4 across specific cancer types. The TCGA datasets used for correlation analysis included 1104 breast cancer (BRCA), 379 ovarian cancer (OV), and 182 pancreatic adenocarcinoma (PAAD) samples. The GSE240671 dataset [56] was downloaded from the GEO database (https://www.ncbi.nlm.nih.gov/geo/, accessed on 23 June 2026). All the data were analyzed with R (version 4.1.3).

### 4.4. Antigen Expression Levels on Tumor Cells

Tumor cells were seeded in 6-well plates at a density of 2 × 10^5^ cells per well and cultured overnight under standard conditions (37 °C, 5% CO_2_). Subsequently, the cells were treated for 48 h with 1 nM PTX (paclitaxel, S1150, Selleck Chemicals, Houston, TX, USA), 2 µM GEM (gemcitabine hydrochloride for injection, Hansoh Pharma, Lianyungang, China) or 5 µM CDDP (cisplatin, P4394, Sigma-Aldrich, St. Louis, MO, USA); then, they were resuspended in FACS buffer (ice-cold PBS containing 2% FBS) and incubated with in-house-generated anti-CXCR4 or anti-PD-L1 nanobodies (1 µg/mL) [33]. FITC-labeled anti-His secondary mAbs (AD1.1.10, Invitrogen, Waltham, MA, USA) were used. The samples were washed twice with FACS buffer, and data were acquired on a flow cytometer (CytoFLEX, Beckman Coulter, Brea, CA, USA).

### 4.5. q-PCR

The RNA was extracted from cells using the Ultrapure RNA Kit (CW0581, CWBIO, Beijing, China), and then, cDNA was prepared by RNA reverse transcription with PrimeScript RT Master Mix (RR036, Takara, Kusatsu, Shiga, Japan). qPCR was conducted following the instructions of TB Green Premix Ex Taq (RR420, Takara). The primers in this study were as follows:

β-actin-F: 5′ CACCATTGGCAATGAGCGGTTC 3′.

β-actin-R: 5′ AGGTCTTTGCGGATGTCCACGT 3′.

CXCR4-F: 5′ CTCCTCTTTGTCATCACGCTTCC 3′.

CXCR4-R: 5′ GGATGAGGACACTGCTGTAGAG 3′.

PD-L1-F: 5′ TGCCGACTACAAGCGAATTACTG 3′.

PD-L1-R: 5′ CTGCTTGTCCAGATGACTTCGG 3′.

### 4.6. Cell Viability Assays

Tumor cells were seeded at a density of 5 × 10^3^ cells/well in 96-well plates. After overnight incubation, chemotherapeutic drugs were administered at graded concentrations and the cells were further cultured for 48 h, to determine the half-maximal inhibitory concentration (IC50).

Tumor cells were seeded in 96-well plates at 5 × 10^3^ per well. Following cell adherence, they were treated with 0.1 µM BsNb PX4 for 12 h, followed by the addition of various concentrations of the chemotherapeutic drug for an additional 48 h.

To evaluate whether CXCL12 promotes chemoresistance and whether BsNb PX4 reverses this effect, tumor cells were seeded in 96-well plates (5 × 10^3^/well), serum-starved for 12 h after attachment, and then cultured in medium containing 2% FBS with 200 ng/mL human CXCL12 (SDF-1α, 300-28A-10UG, PeproTech, Cranbury, NJ, USA) and 0.1 µM BsNb PX4 (alone or in combination). After 4 h, chemotherapeutics (1 nM PTX, 2 µM GEM, or 5 µM CDDP) were added and incubated for another 48 h, with CXCL12 and BsNb PX4 maintained throughout.

Cell viability was measured using Cell Counting Kit-8 (CCK-8) assay (CK04, Dojindo, Kumamoto, Japan). OD450 nm were measured with a microplate reader (Infinite M200 pro, Tecan, Männedorf, Switzerland).

### 4.7. Cell Apoptosis and Cycle Assays

The tumor cells were seeded in 6-well plates at 2 × 10^5^ cells/well overnight and then incubated with 0.1 µM BsNb PX4 for 12 h; they were subsequently incubated with PTX (1 nM), GEM (2 µM), or CDDP (5 µM) for 48 h, respectively. After collection, the cells were stained and detected under the instructions of the Annexin V-FITC/PI kit (70-AP101-100, Multisciences Biotech, Hangzhou, China) or cell cycle kit (CCS012, Multisciences Biotech) and analyzed by CytoFLEX cytometry (Beckman Coulter).

### 4.8. In Vitro Cytotoxicity Assays

MDA-MB-231 cells were seeded at 5 × 10^3^ cells/well overnight in RPMI-1640 medium (without phenol red) containing 10% FBS; then, hPBMCs were added at an E/T ratio of 10:1 or 15:1, with 100 IU/mL IL-2 (Huaxin Biotech, Shanghai, China) maintained in the culture. Subsequently, the chemotherapeutic drugs (PTX at 0, 1, 3.3, and 10 nM; CDDP and GEM at 0, 1, 3.3, and 10 µM) were added to the co-culture system, followed by incubation for 24 h. Finally, 0.1 µM BsNb PX4 was administered and incubated for an additional 24 h.

To examine tumor cell lysis, the release of lactate dehydrogenase (LDH) in the co-culture supernatants was measured by a CytoTox 96^®^ NonRadioactive Cytotoxicity Assay Kit (G1780, Promega, Madison, WI, USA). To exclude potential interference from drug-induced LDH release, the following background controls were included in parallel for each experimental condition: tumor cells alone with the corresponding drug treatment, and effector cells alone with the corresponding drug treatment. The percentage of cytotoxicity was calculated as follows:

Cytotoxicity (%) = (experimental LDH release − effector cell LDH release with drug treatment − target cell LDH release with drug treatment)/(target cell maximum LDH release − target cell spontaneous LDH release) × 100%.

Cytokine release levels in cell culture supernatant were quantified using human IFN-γ (DY285B, R&D systems, Minneapolis, MN, USA), TGF-β1 (ab100647, Abcam, Cambridge, UK), IL-6 (EK106HS, Multisciences), IL-2 (DY202, R&D Systems), Granzyme B (EK158, Multisciences), and Perforin (ab46068, Abcam) ELISA kits according to the manufacturers’ instructions.

### 4.9. Flow Cytometry

The hPBMCs were collected from the 96-well co-culture system and resuspended in FACS buffer to generate single-cell suspensions. Surface marker staining was performed using premixed antibodies—PE-conjugated anti-human CD3 (12-0037-42, eBioscience, Waltham, MA, USA), FITC-conjugated anti-human CD4 (555346, BD Biosciences, Franklin Lakes, NJ, USA), BV421-conjugated anti-human CD8 (562428, BD Biosciences), APC-conjugated anti-human CD69 (555533, BD Biosciences), and Percp-eFluorTM710-conjugated anti-human PD-1 (46-9969-42, eBioscience)—with 30 min incubation at 4 °C protected from light. Unbound antibodies were removed through two wash cycles (300× *g*, 5 min) using FACS buffer. For intracellular Foxp3 detection, cells were fixed with the Foxp3 Transcription Factor Fixation/Permeabilization Factor Staining Buffer Set (00-5523-00, eBioscience) for 30 min at room temperature (RT). After permeabilization, non-specific binding was blocked with 2% normal rat serum for 15 min. Intracellular staining was conducted using PE-Cy7-conjugated anti-human Foxp3 antibody (25-4776-41, eBioscience) at manufacturer-recommended concentrations (2 μg/mL) for 45 min at RT. The cells were finally washed with Permeabilization Wash Buffer and resuspended in 200 μL of FACS buffer for acquisition on an LSRFortessa™ flow cytometer (BD Biosciences) within 24 h.

### 4.10. In Vivo Study

All animal experiments were performed in accordance with the guidelines of the Institutional Animal Care and Use Committee (IACUC) of Shanghai Jiao Tong University (Shanghai, China). Mice aged between 6 and 8 weeks when the experiments commenced were procured from Charles River Laboratories (Beijing, China), and housed in a specific pathogen-free room with a controlled environment (temperature and humidity). Upon their arrival, the animals underwent a 1-week acclimatization period to adapt to their new environment. Continuous daily health monitoring was conducted throughout the study.

#### 4.10.1. Pharmacokinetics Analysis

Ten BALB/c female mice were divided into two subgroups (*n* = 5), and blood samples were collected alternately. A single dose of 5 mg/kg BsNb PX4 was administered via the tail vein, and blood was collected from the cheek pouch at 5 min, 15 min, 30 min, 1 h, 2 h, 4 h, 6 h, 8 h, 12 h, and 24 h post-injection. Concentrations of BsNb PX4 in the plasma were measured using a His Tag ELISA Detection Kit (L00436, GenScript, Nanjing, China) according to the manufacturer’s instructions. Pharmacokinetic parameters were calculated using the PK Solver 2.0.

#### 4.10.2. In Vivo Biodistribution

For in vivo biodistribution imaging, BsNb PX4 was labeled with Cy5.5. Briefly, 0.5 mg of Cy5.5 NHS ester (N783250, Macklin, Shanghai, China) was added to 1 mg of BsNb PX4 in 1 mL of 100 mM sodium bicarbonate buffer (pH 8.0). After incubation at 4 °C for 8 h with gentle shaking, free Cy5.5 molecules were removed by repeated dialysis. The final labeling mole ratio of Cy5.5 to BsNb PX4 was 3.97. MDA-MB-231 cells (5 × 10^6^ cells per mouse) were subcutaneously injected into female BALB/c nude mice (*n* = 5). When tumor volume reached approximately 100 mm^3^, Cy5.5-labeled BsNb PX4 was administered at a single dose of 1 mg/kg via tail vein injection. Fluorescence images were taken on an IVIS Spectrum system (PerkinElmer, Waltham, MA, USA) at 0 h, 0.5 h, 4 h, 24 h, and 72 h. Subsequently, the mice were sacrificed and major organs including the tumor, liver, kidney, lung, and spleen were collected for immunohistochemistry staining with anti-His tag antibodies (10001-0-AP, Proteintech, Rosemont, IL, USA) to assess the tissue distribution of BsNb PX4.

#### 4.10.3. Tumor/hPBMC Co-Grafting Setting

MDA-MB-231 cells (5 × 10^6^ cells) were mixed with hPBMCs (3 × 10^6^ cells); then, an equal volume of ice-cold Matrigel (356234, Corning Incorporated, Corning, NY, USA) was mixed with the cells. The tumor cell/hPBMC mixture was implanted into female NOD/SCID mice in the armpit via subcutaneous injection (s.c.). The mice were randomly divided into four groups based on body weight: PBS, BsNb PX4, paclitaxel, and combination of paclitaxel and BsNb PX4. BsNb PX4 was administered intravenously (i.v.) via the tail vein at dose of 1 mg/kg, totaling 8 doses over a 24-day treatment period, while paclitaxel was administered intraperitoneally (i.p.) at dose of 10 mg/kg, every 6 days for a total of four doses. The same dose schedules were applied in the combination studies. Tumor volume (TV) was calculated according to the following formula:

TV (mm^3^) = (Length × Width^2^)/2.

To preliminarily assess the safety profiles of the combined treatment, we monitored body weight and measured six serum biochemical markers using the Mindray Chemistry Analyzer BS360S (Shenzhen, China)—alanine aminotransferase (ALT), aspartate aminotransferase (AST), creatinine (CREA-S), urea (UREA), uric acid (UA), and total protein (TP II)—to evaluate hepatorenal toxicity.

#### 4.10.4. Tumor Model with Intraperitoneal Transfer of hPBMCs

Female NOG mice received subcutaneous implants of MDA-MB-231-Luc cells (2 × 10^6^ cells per mouse). Each mouse was injected intravenously with 5 × 10^6^ hPBMCs via the tail vein after a 5-day interval following subcutaneous implant. Randomization was performed based on body weight, tumor volume, and tumor bioluminescence value. On the third day following the hPBMC injection, BsNb PX4 was dosed at 1 mg/kg (i.v.), totaling seven doses during the treatment period; in parallel, paclitaxel (10 mg/kg, i.p.) or gemcitabine (30 mg/kg, i.p.) was administered every six days for a total of three doses, respectively; the mice in combination therapy groups were administered with the same dosages. The mice were injected with D-Luciferin potassium salt (ST196, Promega) for measuring tumor bioluminescence using an IVIS Spectrum system with the Living Image software (version 4.4, PerkinElmer), while tumor growth was monitored using bioluminescence imaging and a digital caliper.

At the end of the in vivo experiments, blood samples were collected for a routine blood test, including platelets (PLTs), red blood cells (RBCs), white blood cells (WBCs), and hemoglobin (HGB), and measured by a Sysmex Hemostasis Analyzer XN-1000V (Kobe, Japan). The tumors were stripped and weighted under nuclease-free conditions.

### 4.11. Hematoxylin–Eosin, Immunofluorescence and Immunohistochemistry Staining

Tumors were fixed in 4% paraformaldehyde (G1101, Servicebio, Wuhan, China) for 24 h, and embedded in paraffin.

For hematoxylin–eosin (HE) staining, the tumor sections were counterstained with HE (G1076, Servicebio), and the slides were dehydrated in ethanol and cleared with xylene. Images of the stained slides were acquired by an OLYMPUS BX53 microscope (Olympus, Tokyo, Japan). Mouse major organ tissue pathologies were evaluated by HE staining following standard procedures.

For immunofluorescence (IF) staining, the tumor sections were cut, and then, deparaffinization, heat antigen retrieval, and endogenous peroxidase blocking were conducted. Then, the sections were blocked with 5% BSA at room temperature for 1 h and incubated with anti-CD8 alpha antibody (ab245118, Abcam) at a 1/500 dilution in the dark for 1 h. HRP-conjugated goat anti-rabbit IgG polyclones (PV-6001, Zsbio, Beijing, China) as secondary antibody were used with Opal™690 reagent (Akoya Biosciences, Marlborough, MA, USA). After being immunolabelled, the sections were washed and stained with DAPI (D9542, Sigma) for 10 min at room temperature, and the images were visualized under a NIKON ECLIPSE C1 microscope (NIKON, Tokyo, Japan). Additionally, a TNBC tissue microarray (ZL-Brc3N961, Zhuoli Biotechnology Co., Shanghai, China) was used, as well as IF staining for PD-L1 (13684, Cell Signaling Technology, Danvers, MA, USA) and CXCR4 (HA722304, HUABIO, Hangzhou, China).

For immunohistochemistry (IHC) staining, the tumor sections were incubated with primary antibodies against human CD3 (60181-1-Ig, Proteintech) or human CD8 (ab245118, Abcam, UK). Antigen detection was achieved with an HRP-conjugated secondary antibody and DAB chromogen (P0203, Beyotime, Shanghai, China), with subsequent hematoxylin counterstaining. Finally, slides were scanned using the Pannoramic SCAN system (3DHISTECH, Budapest, Hungary).

### 4.12. RNA Sequencing

Total mRNA was extracted from the stripped tumors of the MDA-MB-231/hPBMC co-grafting model, and the purity and concentration of the extracted mRNA were measured using a NanoDrop 2000 spectrophotometer (Thermo Fisher Scientific). cDNA library construction, sequencing using NovaSeq X Plus (Illumina, San Diego, CA, USA), and alignment to the human genome assembly (GRCh38) using HISAT2 were conducted by Majorbio Bio-pharm Technology Co., Ltd. (Shanghai, China). Differentially expressed genes (DEGs) were identified based on the criteria of a *p*-value of 0.05 and a log2 (fold change, FC) of 2. Kyoto Encyclopedia of Genes and Genomes (KEGG) and Gene Set Enrichment Analysis (GSEA), as well as hierarchical clustering analysis, were performed on the Majorbio Cloud online platform. The abundance of immune cells was analyzed by Immune Cell Abundance Identifier (ImmuCellAI, https://guolab.wchscu.cn/ImmuCellAI/#!/, accessed on 23 June 2026).

### 4.13. Statistical Analysis

GraphPad Prism (version 8.0, GraphPad Software, San Diego, CA, USA) was used for visualizing the data, which are summarized as the mean ± standard deviation (SD). Statistical analysis was based on Student’s *t*-test or ANOVA, and statistical significance was set at **** *p* < 0.0001, *** *p* < 0.001, ** *p* < 0.01, and * *p* < 0.05.

## 5. Conclusions

In this study, we designed and validated BsNb PX4, a bispecific nanobody targeting CXCR4 and PD-L1, which features efficient tumor penetration and prolonged tumor retention, directly sensitizes tumor cells to paclitaxel, and effectively reverses the immunosuppressive tumor microenvironment in TNBC models, thereby amplifying the chemotherapeutic response. Furthermore, its combination with other chemotherapeutic agents demonstrated significantly enhanced antitumor efficacy across multiple solid tumor models, highlighting its broad applicability and positioning it as a promising candidate for clinical translation.

## Data Availability

The original contributions presented in this study are included in the article and Supplementary Material. Further inquiries can be directed to the corresponding authors.

## Supplementary Materials

The following supporting information can be downloaded at https://www.mdpi.com/article/10.3390/ph19081288/s1, Figure S1: IHC staining of the tumor, liver, kidney, lung, and spleen sections with anti-His tag antibodies to detect the localization of BsNb PX4; Figure S2: The surface expression of PD-L1 and CXCR4 on tumor cells following chemotherapy treatment was assessed by flow cytometry; Figure S3: Box-plot of RNA sequencing results was sourced from GSE240671; Figure S4: Effect of different chemotherapeutic drugs on tumor cell viability; Figure S5: IL-6 secretion in the hPBMC-tumor cell co-culture system; Figure S6: In vivo safety evaluation of different treatments in the MDA-MB-231/hPBMC co-grafting model; Figure S7: BsNb PX4 combined with chemotherapy shows efficacy and safety in MDA-MB-231-luc xenograft model with intravenous transfer of hPBMCs; Table S1: The pharmacokinetics of BsNb PX4; Table S2: Summary of in vitro cytotoxicity (IC50) of chemotherapy drugs.

## Abbreviations

The following abbreviations are used in this manuscript:

TNBC	Triple-Negative Breast Cancer
TME	Tumor Microenvironment
PD-1	Programmed Cell Death-1
PD-L1	Programmed Death-Ligand 1
CXCR4	C-X-C Chemokine Receptor 4
CXCL12	C-X-C Motif Ligand 12
ECM	Extracellular Matrix
CAFs	Cancer-Associated Fibroblasts
EMT	Epithelial–Mesenchymal Transition
hPBMCs	Human Peripheral Blood Mononuclear Cells
Tregs	Regulatory T Cells
ER	Estrogen Receptor
PR	Progesterone Receptor
HER2	Human Epidermal Growth Factor Receptor 2
pCR	Pathological Complete Response
PFS	Progression-Free Survival
MDSCs	Myeloid-Derived Suppressor Cells
TAMs	Tumor-Associated Macrophages
MMPs	Matrix Metalloproteinases
CSCs	Cancer Stem Cells
Nbs	Nanobodies
VHH	Variable Domain of Heavy Chain of Heavy-Chain Antibody
SDF-1α	Stromal Cell-Derived Factor 1 Alpha
α-SMA	Alpha Smooth Muscle Actin
AFM	Atomic Force Microscope
DLS	Dynamic Light Scattering
PDI	Polydispersity Index
TCGA	The Cancer Genome Atlas
CCLE	Cancer Cell Line Encyclopedia
PTX	Paclitaxel
GEM	Gemcitabine
CDDP	Cisplatin
LDH	Lactate Dehydrogenase
ICD	Immunogenic Cell Death
DAMPs	Damage-Associated Molecular Patterns
ROI	Region-Of-Interest
HE	Hematoxylin–Eosin
IF	Immunofluorescence
IHC	Immunohistochemistry
ALT	Alanine Aminotransferase
AST	Aspartate Aminotransferase
CREA-S	Serum Creatinine
UA	Uric Acid
TP	Total Protein
GVHD	Graft-Versus-Host Disease
WBC	White Blood Cell
HGB	Hemoglobin
PLT	Platelet
RBC	Red Blood Cell
DEGs	Differentially Expressed Genes
KEGG	Kyoto Encyclopedia of Genes and Genomes
GSEA	Gene Set Enrichment Analysis
NES	Normalized Enrichment Score
TILs	Tumor-Infiltrating Lymphocytes
ICI	Immune Checkpoint Inhibitor
HCC	Hepatocellular Carcinoma
FACS	Fluorescence-Activated Cell Sorting
IC50	Half-Maximal Inhibitory Concentration
RT	Room Temperature
