# Supplementary material for: A CXCR4/PD-L1 Bispecific Nanobody Engineered for Tumor Microenvironment Retention Mediates Sustained Synergy with Chemotherapy via Remodeling Immunity in TNBC
Source: Pharmaceuticals (Basel). 2026 Aug 14;19(8):1288. doi: 10.3390/ph19081288 (PMC13516925; doi:10.3390/ph19081288)
Supplement: Supplementary file 1 [file pharmaceuticals-19-01288-s001.zip › pharmaceuticals-4425514-supplementary.pdf]

Supplemental Material: A CXCR4/PD-L1 Bispecific Nanobody Engineered for Tumor Microenvironment Retention Mediates Sustained Synergy with Chemotherapy via Remodeling Immunity in TNBC

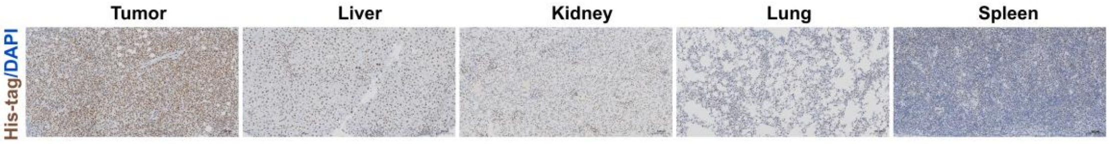

Figure S1. IHC staining of tumor, liver, kidney, lung, and spleen sections with anti-His tag antibodies to detect the localization of BsNb PX4.

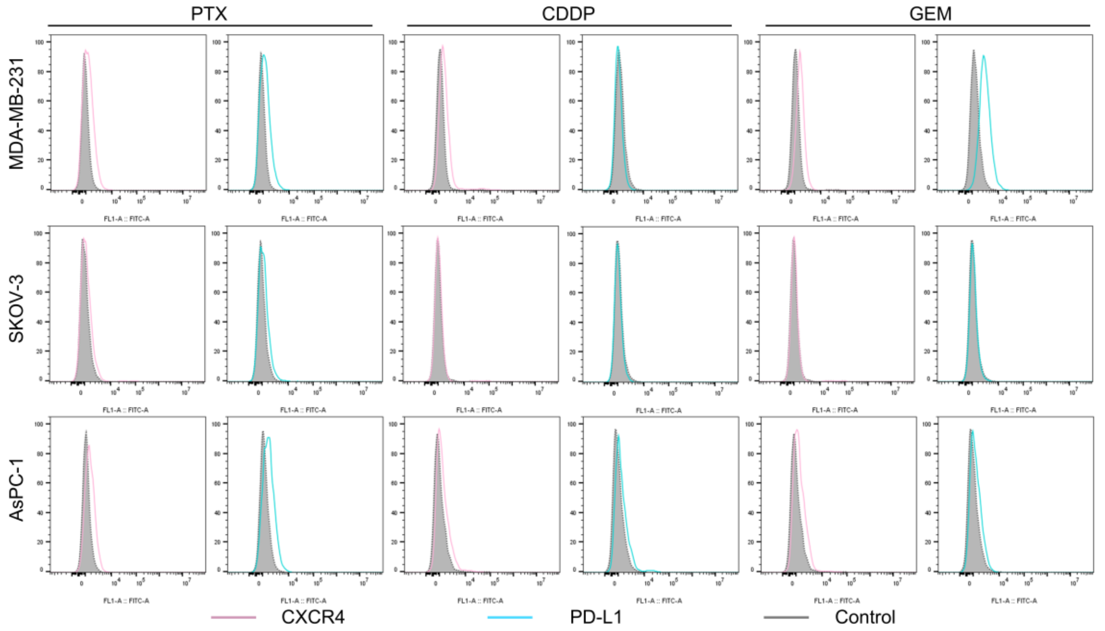

Figure S2. The surface expression of PD-L1 and CXCR4 on tumor cells following chemotherapy treatment was assessed by flow cytometry.

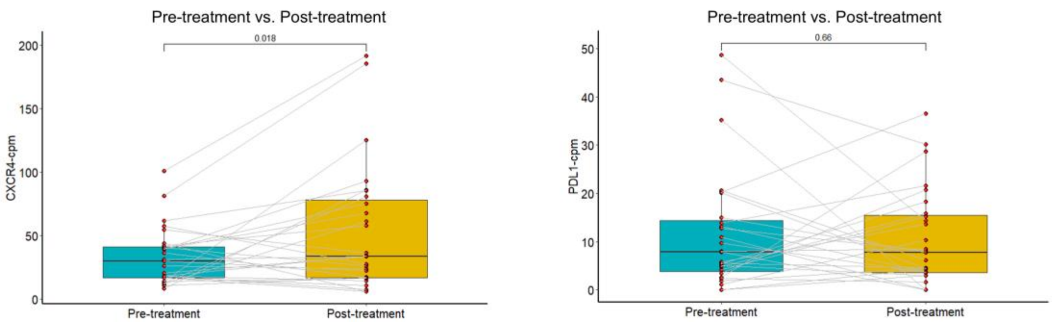

Figure S3. Box-plot of RNA sequencing results was sourced from GSE240671. The expression of CXCR4 and PD-L1 in patient data before and after drug administration was determined by a paired sample t-test. The dataset includes multiple breast cancer subtypes (27 paired samples).

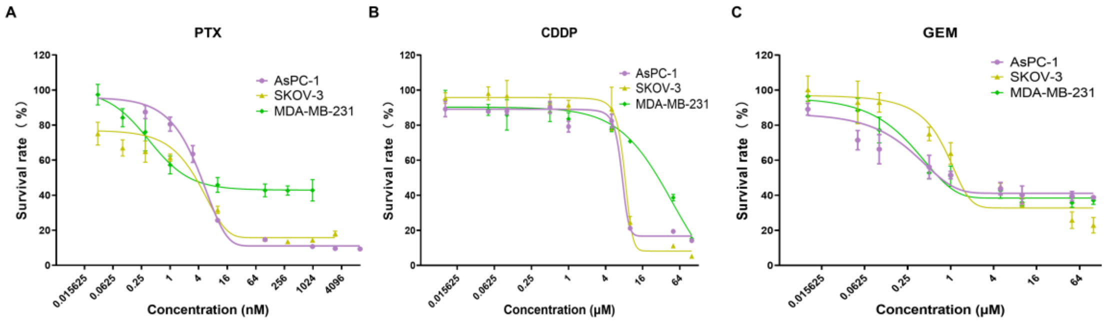

Figure S4. Effect of different chemotherapeutic drugs on tumor cell viability. (**a**) Evaluation of tumor cell viability after PTX treatment 48 h (n=3). (**b**) Evaluation of tumor cell viability after CDDP treatment 48 h (n=3). (**c**) Evaluation of tumor cell viability after GEM treatment 48 h (n=3).

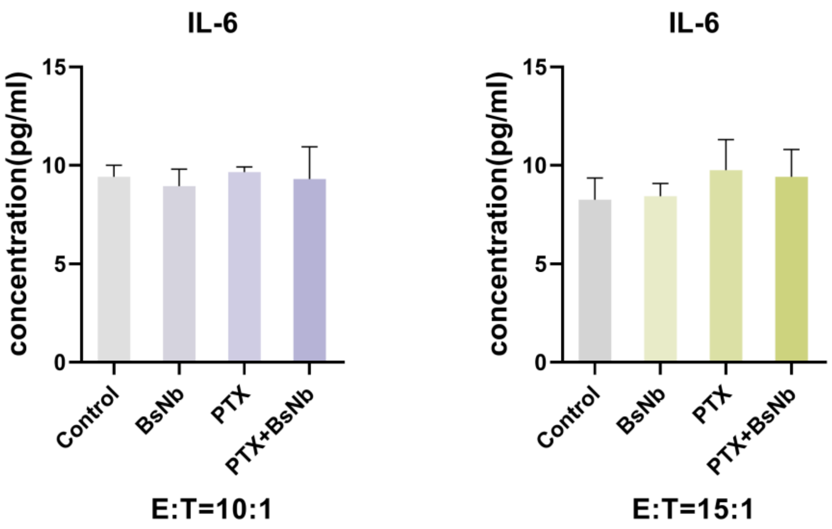

Figure S5. IL-6 secretion in the hPBMC-tumor cell co-culture system. Supernatant IL-6 levels were measured by ELISA. Data are presented as mean ± SD (n = 3).

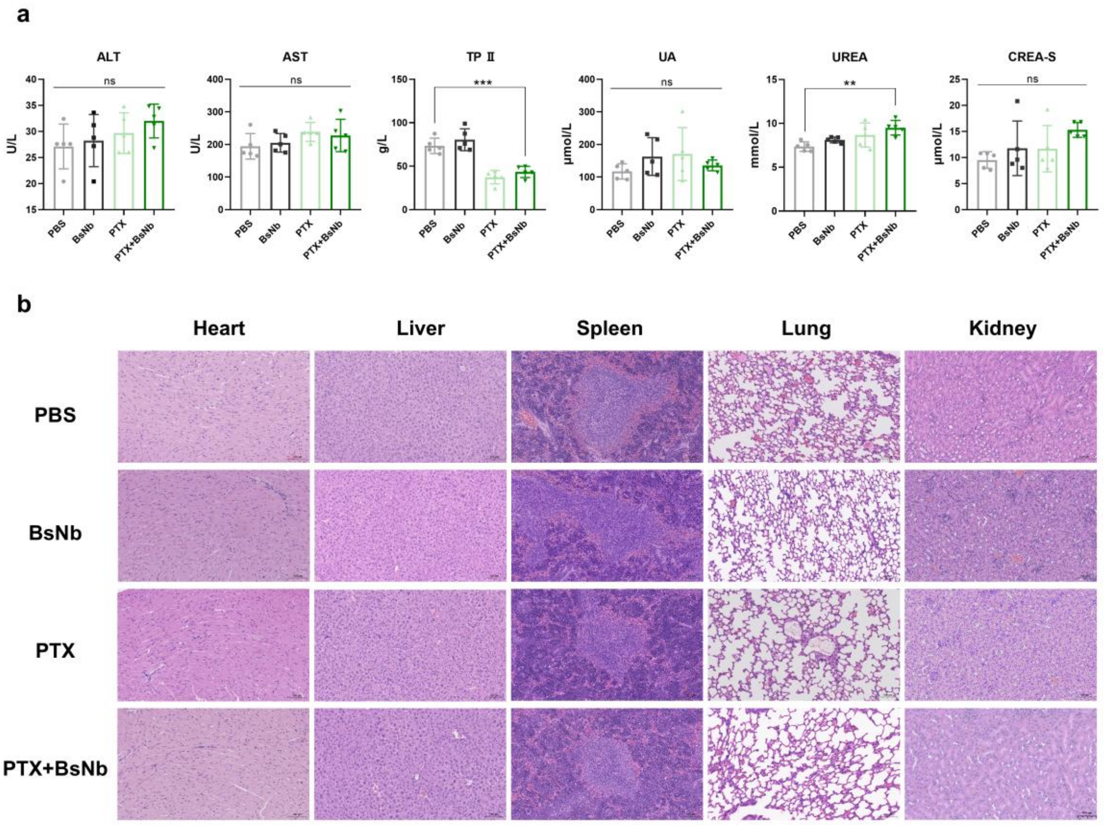

Figure S6. In vivo safety evaluation of different treatments in the MDA-MB-231/hPBMCs co-grafting model. **(a)** Liver and kidney biochemical indexes of mice in different treatment groups at the end of the experiment. Significance determined by one-way ANOVA with Tukey's test (n=5, ****P* < 0.001, ***P* < 0.01, ns, no significance). (**b**) HE staining of organs in MDA-MB-231/hPBMCs co-grafting model.

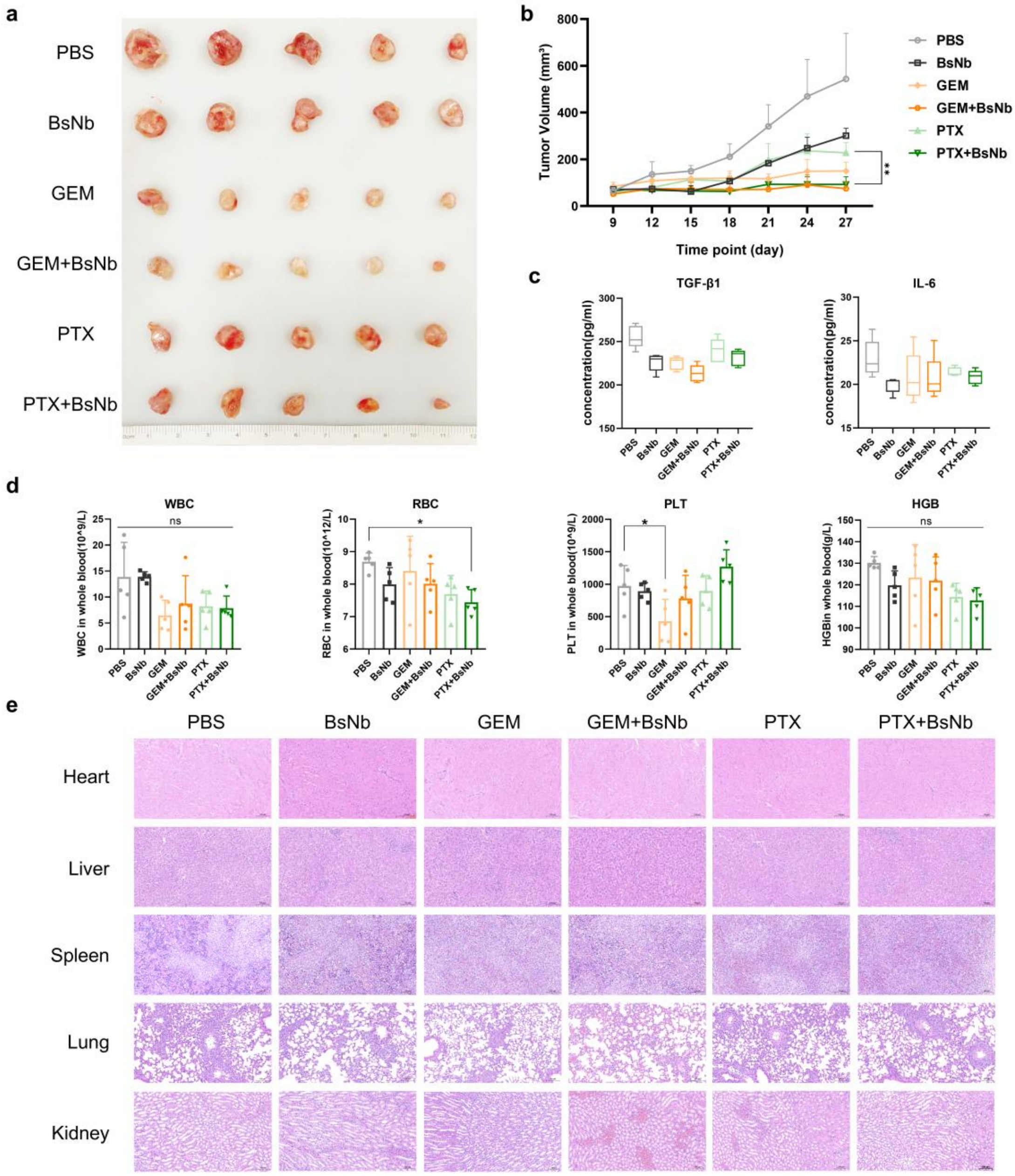

Figure S7. BsNb PX4 combined with chemotherapy shows efficacy and safety in MDA-MB-231-luc xenograft model with intravenous transfer of hPBMCs. (**a**) All tumor masses were photographed. (**b**) Tumor volume change curves of different treatment groups. Data are presented as mean ± SD, and statistical significance was determined by Two-way ANOVA. n=5, ***P* < 0.01. (**c**) TGF-β1 and IL-6 detection in mice serum at the end of the experiment. (**d**) Blood routine indexes of mice in different treatment groups at the end of the experiment. Significance determined by one-way ANOVA with Tukey's test, **P* < 0.05, ns, no significance. (**e**) HE analysis of organs (heart, liver, lung, spleen, and kidney) sections.

Table S1. The pharmacokinetics of BsNb PX4.

Parameter	BsNb PX4
Dose (5 mg/kg)	5.00
$T_{1/2}$ (h)	2.47
C_{max} (mg/mL)	1.37
AUC 0-t (mg/mL × h)	2.80

Table S2. Summary of in vitro cytotoxicity (IC50) of chemotherapy drugs.

Drugs	Cell lines	IC50	95% Confidence Interval	R^2
GEM	AsPC-1	3.192 μM	1.993 to 5.306 μM	0.8987
	SKOV-3	4.338 μM	3.339 to 5.673 μM	0.9508
	MDA-MB-231	3.235 μM	1.851 to 5.939 μM	0.8771
CDDP	AsPC-1	7.273 μM	6.160 to 8.562 μM	0.8528
	SKOV-3	7.871 μM	7.299 to 8.483 μM	0.9601
	MDA-MB-231	21.49 μM	15.56 to 29.77 μM	0.9025
PTX	AsPC-1	3.583 nM	2.903 to 4.435 nM	0.9867
	SKOV-3	1.779 nM	0.7473 to 4.657 nM	0.9199
	MDA-MB-231	0.3540 nM	0.2349 to 0.5410 nM	0.9563
